# Interplay between traumatic brain injury and intimate partner violence: data driven analysis utilizing electronic health records

**DOI:** 10.1186/s12905-020-01104-4

**Published:** 2020-12-07

**Authors:** Larry Y. Liu, William S. Bush, Mehmet Koyutürk, Günnur Karakurt

**Affiliations:** 1 Systems Biology and Bioinformatics Program, Case Western Reserve University, Cleveland, OH USA; 2 Department of Population and Quantitative Health Sciences, Case Western Reserve University, Cleveland, OH USA; 3 Department of Computer and Data Sciences, Case Western Reserve University, Cleveland, OH USA; 4 Department of Psychiatry, Case Western Reserve University, Cleveland, OH USA

**Keywords:** Electronic health records, Intimate partner violence, Traumatic brain injury, Domestic violence, Cohort study, Blunt force, Data mining

## Abstract

**Background:**

It is estimated that a majority of intimate partner violence (IPV) victims suffer from blunt force to the head, neck and the face area. Injuries to head and neck are among the major causes for traumatic brain injury (TBI).

**Methods:**

In this interdisciplinary study, we aimed to characterize the key associations between IPV and TBI by mining de-identified electronic health records data with more than 12 M records between 1999 to 2017 from the IBM *Explorys* platform. For this purpose, we formulated a data-driven analytical framework to identify significant health correlates among IPV, TBI and six control cohorts. Using this framework, we assessed the co-morbidity, shared prevalence, and synergy between pairs of conditions.

**Results:**

Our findings suggested that health effects attributed to malnutrition, acquired thrombocytopenia, post-traumatic wound infection, local infection of wound, poisoning by cardiovascular drug, alcoholic cirrhosis, alcoholic fatty liver, and drug-induced cirrhosis were highly significant at the joint presence of IPV and TBI.

**Conclusion:**

To develop a better understanding of how IPV is related to negative health effects, it is potentially useful to determine the interactions and relationships between symptom categories. Our results can potentially improve the accuracy and confidence of existing clinical screening techniques on determining IPV-induced TBI diagnoses.

## Background

Millions of women are affected each year by intimate partner violence (IPV) [1]. IPV can be in the form of physical violence, sexual violence, or psychological harm. These harmful acts could be carried out by a current partner, spouse, or a former partner [2]. According to the CDC data, approximately one-quarter of women experience severe physical violence by an intimate partner during their lifetime, such as being slammed, hit, or beaten [1]. Depending on the severity of the perpetrated violence, IPV is often linked to health problems ranging from minor cuts to severe health consequences, and death [2, 3]. In addition to acute conditions, prior studies have shown that IPV complications can be chronic. Victims often suffer from various neurological symptoms, mental health and substance disorders, gastrointestinal problems, and chronic pain [3, 4]. Furthermore, injury attained from IPV is most likely to lead to traumatic brain injury (TBI) [5, 6]. Emergency room records show that 38% of IPV victims who received emergency medical treatment exhibit significant head, neck, and facial injuries [7]. It is estimated that this number is much higher, with 60% to 92% of surviving female IPV victims receiving facial, head, or neck strangulation injuries [8].

There has been increasing awareness of IPV victims’ suffering from TBI [9]. TBI can be described as changes in brain function or detecting any brain pathology due to an external force [10]. A recent review found that head trauma and neck strangulations caused by perpetrators are prevalent enough to be classified as an injury mechanism for TBI [7, 11]. Neuronal damage is typically observed after trauma to the head, which is frequently trailed by ancillary events disruptive to the neurons and neuronal systems [12]. These ancillary effects might be linked to later structural changes, metabolic dysfunction, or cell death [12]. In addition, it is frequently observed that during the process of closed head injuries, rather than the site of skull impact, the injury to the opposite side of the impact results in more severe brain contusion. This is called contrecoup brain injury, while coup injury is defined as the location of the initial impact which tends to be less severe [13]. Strangulation, on the other hand, is a process of asphyxia that comprises occlusion of airways, occlusion of neck vessels, compression of the carotid arteries resulting in hypoxia, cerebral ischemia, carotid sinus reflex, or cardiac arrest [14]. Strangulation is observed as part of severe fatal and non-fatal violence [15]. All these changes might result in short or long term cognitive, behavioral, emotional as well as physical challenges [16].

IPV victims suffering from TBI complications exhibit additional adverse health effects. A systematic review notes that IPV victims who suffer from anxiety, depression, dizziness, and headaches show symptoms that are consistent with a post-concussive syndrome or lingering mild TBI [7, 8]. This suggests that, aside from PTSD and stress, these additional symptoms may hint at signs of brain injury [7, 8]. A 2003 study on assessing brain injury on female victims reveals that the severity of abuse is negatively correlated with cognitive function and positively correlated with brain injury [11]. Furthermore, neuro-anatomical studies reveal that women who experience IPV exhibit symptoms of injuries to four neural regions that affect behavior and decision-making skills: The amygdala, the prefrontal cortex, the hypothalamus, and the hippocampus [7, 17–19].

Currently established IPV screening processes are limited and they are not fully reliable as they often rely on surveys and self-reports [20]. Most victims would not disclose abuse to clinicians due to the negative stigma associated with IPV [7, 21, 22]. For similar reasons, medical providers can also be reluctant to screen for IPV. Screening IPV victims for TBI complication is even more difficult since most TBI screening tests are designed for victims of various environmental accidents, making it difficult to reveal and signs of abuse [8].

### Objective

In this study, we develop a data-driven method that utilizes electronic health records (EHR) data to systematically investigate the relationship between IPV and TBI. EHR databases contain records of clinicians’ diagnoses and findings on patient visits. Therefore, EHR data is different from self-report or survey-based data, in that the information is provided by a third party who is an expert [23]. EHR databases utilize Systematized Nomenclature of Medicine Clinical Terms (SNOMED-CT) and International Classification of Diseases (ICD-9) as well [23], making it easier to accurately identify and associate diagnoses [23]. SNOMED-CT is an extensive standardized clinical terminology, which presents a language for clinical content and improves communication for clinical documentation, sharing, and exchange of information. It is also hosted by the BioOntology Portal of the National Center for Biomedical Ontology. Similarly, ICD is also a system with alpha-numeric diagnosis codes and assists in classifying signs, symptoms, diseases, conditions, and procedures. ICD codes are usually automatically mapped based on SNOMED-CT in EHR systems. Finally, EHR databases provide a huge sample to work with, often containing over tens of millions of patient records from multiple hospitals with broad geographical distribution [9].

Motivated by the opportunities offered by the nature of EHR data, we mined datasets extracted from an EHR database, for the purpose of finding *key adverse health effects that are associated with (i.e., either arise from or contribute to) the interplay between IPV and TBI*. The results of this analysis can be useful in verifying and interpreting the health effects discovered by observational studies. New findings can also inform the development of novel hypotheses, and hence the design and implementation of new observational studies. To systematically investigate the adverse health correlates of IPV and TBI, we explore three questions:

- Research Question 1: How many terms are *shared* between patients who are exposed to IPV and patients who are diagnosed with TBI? What are these terms?
- Research Question 2: How many terms are *commonly prevalent* in both patient populations with IPV and patient populations with TBI? What are these terms?
- Research Question 3: How many terms are *synergistically prevalent* in patients who are exposed to IPV and diagnosed with TBI? What are these terms?

Here, a *term* refers to a finding or diagnosis that is uniquely defined by *Explorys* using SNOMED-CT. The first question aims to identify all terms that are *seen alongside IPV and TBI*. The second question aims to identify terms that are *associated with both IPV and TBI*, in terms of significantly higher frequency in patients with IPV and TBI as compared to the overall population. The third question seeks to identify terms that are not necessarily associated with either IPV or TBI but are *associated with the existence of both IPV and TBI* in a patient.

## Methods

### Database and queries

To generate patient cohorts and extract frequencies of findings, we use the *Explorys* EHR Platform by IBM Watson Health [24]. The IBM *Explorys* platform is comprised of IBM *Explorys* Cohort Discovery, IBM *Explorys* Therapeutic Datasets, and IBM *Explorys* Virtual Workbench, and provides over 54 M unique patient records from 344 K unique providers nationally and from 23 integrated healthcare systems [24]. IBM *Explorys* Cohort Discovery can be accessed through a web browser and it can be used to search and browse patient populations, analyze relationships between cohorts, define temporal events in clinical records, and understand historical trends. Researchers from diverse disciplines have reliably utilized the data provided by *Explorys* database to identify patterns in diseases, treatments, and outcomes [25, 26].

We query *Explorys* in order to identify the terms that are associated with IPV and/or TBI. Each query executed in *Explorys* extracts the frequencies of diagnostic terms in a cohort. A *cohort,* in this case, is the set of all records that satisfy a set of constraints on their attributes. These constraints can be defined on demographic (e.g., all-female patients or all patients above the age of 18), as well as medical attributes (e.g. all patients that contain the finding “traumatic brain injury”). In this study, all queries contain the constraint of being female and being between the ages of 18 and 65 (in the age field) [7]. In addition to these constraints, we define a “cohort” specific to a condition (e.g., IPV or TBI) by adding a constraint that requires the existence of a term (e.g., “domestic abuse” for IPV, “traumatic brain injury” for TBI). Once a query is defined in terms of these constraints, *Explorys* constructs the respective cohort as the set of records that satisfy all the constraints. For each cohort, we download a table from *Explorys*, which includes the descriptive statistics for demographics, as well as all the frequencies of the terms that exist in the cohort. The *frequency of a term in a cohort* is the number of records in the cohort that reference the respective term.

We use the SNOMED-CT term “domestic abuse” to define the IPV cohort. In the SNOMED-CT ontology, the parent term of “domestic abuse” is “abuse”, and its children are “abuse of partner”, “domestic abuse of adult”, “domestic emotional abuse”, “domestic sexual abuse”, and “domestic violence”. In the SNOMED-CT ontology, the parent term of “elderly person maltreatment” is also “Abuse” and its children are “abandonment of elderly person”, “deprivation of nourishment of an elderly person”, “emotional abuse of elderly person”, “neglect of elder”, and “physical abuse of an elderly person”. Since the term “abuse” is too general to describe IPV, and since it also included abuse of elderly persons, “domestic abuse” stands out as the most a descriptive term for IPV in the SNOMED-CT ontology.

### Control cohorts and associations

To account for any confounding bias, we establish control cohorts for our study [27]. The use of control cohorts also enables assessing the statistical significance of the terms that are found to be associated with intimate partner violence (IPV) and traumatic brain injury (TBI). For this study, we utilize two sets of control groups: acute conditions and accident-related incidents.

The acute conditions control group represents conditions that are commonly encountered in the overall population, are not reported to have an association with IPV or TBI, are not chronic, and are not likely complications from other underlying causes. The idea behind using these control cohorts is that these conditions likely do not have any association with IPV, therefore, the patterns identified on these cohorts are likely representative of noise and any source of bias in the data. As such, we select appendicitis (App), tonsillitis (Ton), and gallstones (Gall) as the three acute conditions used to generate control cohorts. Our literature search indicated no specific associations between these conditions and IPV. Since IPV is prominent in the general population and these are common conditions, we expect a sizable overlap between the IPV cohort and each of these control cohorts. Therefore, the patterns identified in these cohorts may reveal noise and any source of bias in the data. However, it is possible that previously unreported comorbidities exist between IPV and these conditions.

The second set of control groups comprises accident-related incidents. Specifically, we select motor vehicle accidents (MVA), sports-related accidents (SA), and falling off stairs (FoS) as the conditions to represent the accident-related control group. These conditions are chosen as likely correlates of TBI, i.e., TBI may stem from any of these three types of incidents. Furthermore, while these conditions are not specifically related to IPV, it is possible that they used as a decoy for IPV, because of possible legal consequences of or the stigma associated with IPV. It is also possible that MVA and FoS are associated with IPV as vehicles can be used as weapons by perpetrators and violence at home can result in falling off stairs. For these reasons, the accident-related control group enables dissection of the relationship between IPV and TBI in the context of different types of accidents/incidents.

### Querying and cohort formation

We utilize the “*Explorys* Cohort Discovery” tool to create cohorts by running separate queries on *Explorys*. The respective queries are as follows:

#### IPV Cohort (8140 records)

All records containing the term “domestic violence” (IPV) in the “diagnosis or findings” field.

#### TBI Cohort (116,600 records)

All records containing the term “traumatic brain injury” (TBI) in the “diagnosis or findings” field.

#### IPV ∩ TBI Cohort (610 records)

All records containing both the term “traumatic brain injury” (TBI) and the term “domestic violence” (IPV) in the “diagnosis or findings” field.

#### X cohort

All records containing the term “X” in the “diagnosis or findings” field (where X represents each of the 6 control conditions). MVA: 516,040 records, SA: 101,570 records, FoS: 121,420 records, App: 75,600 records, Ton: 238,220 records, Gall: 319,320 records.

#### IPV ∩ X cohort

All records containing both the term “X” and the term “domestic violence” (IPV) in the “diagnosis or findings” field (where X represents each of the 6 control conditions). IPV ∩ MVA: 1,220 records, IPV ∩ SA: 190 records, IPV ∩ FoS: 690 records, IPV ∩ App: 90 records, IPV ∩ Ton: 280 records, IPV ∩ Gall: 450 Records.

#### Background Cohort (12,684,250 records)

All records that satisfy the demographic constraints noted above.

In total, we obtained 16 cohorts through these queries. The cohort sizes and the number of terms attributed to each of these cohorts are shown in Table 1. All queries were run on April 28th, 2017 with *Explorys* marking the last revision of the database on April 24th, 2017. The time frame of records ranges from 1999 to April 24th, 2017.

**Table 1:** Cohort size and the number of terms for each cohort that was constructed by querying *Explorys*

	Cohorts of interest					
Query/cohort	IPV		TBI		IPV ∩ TBI	
Cohort size	8140		116,660		610	
Number of terms	5460		11,185		2248	

	Accident-related incidents					
Query/cohort	MVA	IPV ∩ MVA	SA	IPV ∩ SA	FoS	IPV ∩ FoS
Cohort Size	516,040	1220	101,570	190	121,520	690
Number of Terms	11,185	3191	9547	1223	11,185	2655

	Acute conditions					
Query/cohort	App	IPV ∩ App	Ton	IPV ∩ Ton	Gall	IPV ∩ Gall
Cohort size	75,600	90	238,220	280	319,320	450
Number of terms	9755	714	11,135	1446	11,185	2107

	Background (Overall population of females 18–65 years of age)					
Cohort size	12,684,250					
Number of terms	11,185					

*Cohort size* is the number of records that contain the corresponding term as a finding or diagnosis. *Number of terms* is the number of terms that have non-zero frequency in the respective cohort. *IPV* intimate partner violence, *TBI* traumatic brain injury, *MVA* motor vehicle accident, *SA* sports accident, *FoS* falling off stairs, *App* appendicitis, *Ton* tonsillitis, *Gall* Gallstones

### Data analysis

#### Identification of shared terms

To determine the set S_TBI_ of shared terms between IPV and TBI cohorts, we identify the terms that have a non-zero frequency in the TBI ∩ IPV cohort. Similarly, for each control cohort X, we compute S_X_ as the set of terms that have a non-zero frequency in the X ∩ IPV cohort. Since the expected number of shared terms between a cohort and the IPV cohort is a function of the number of records in that cohort, we visualize the relationship between the number of records and the number of shared terms by plotting |S_X_| as a function of the number of records in the control cohort (N_X_). We then assess the relationship between |S_TBI_| and N_TBI_ in the context of this visualization.

#### Identification of commonly prevalent terms

**Fig. 1:**
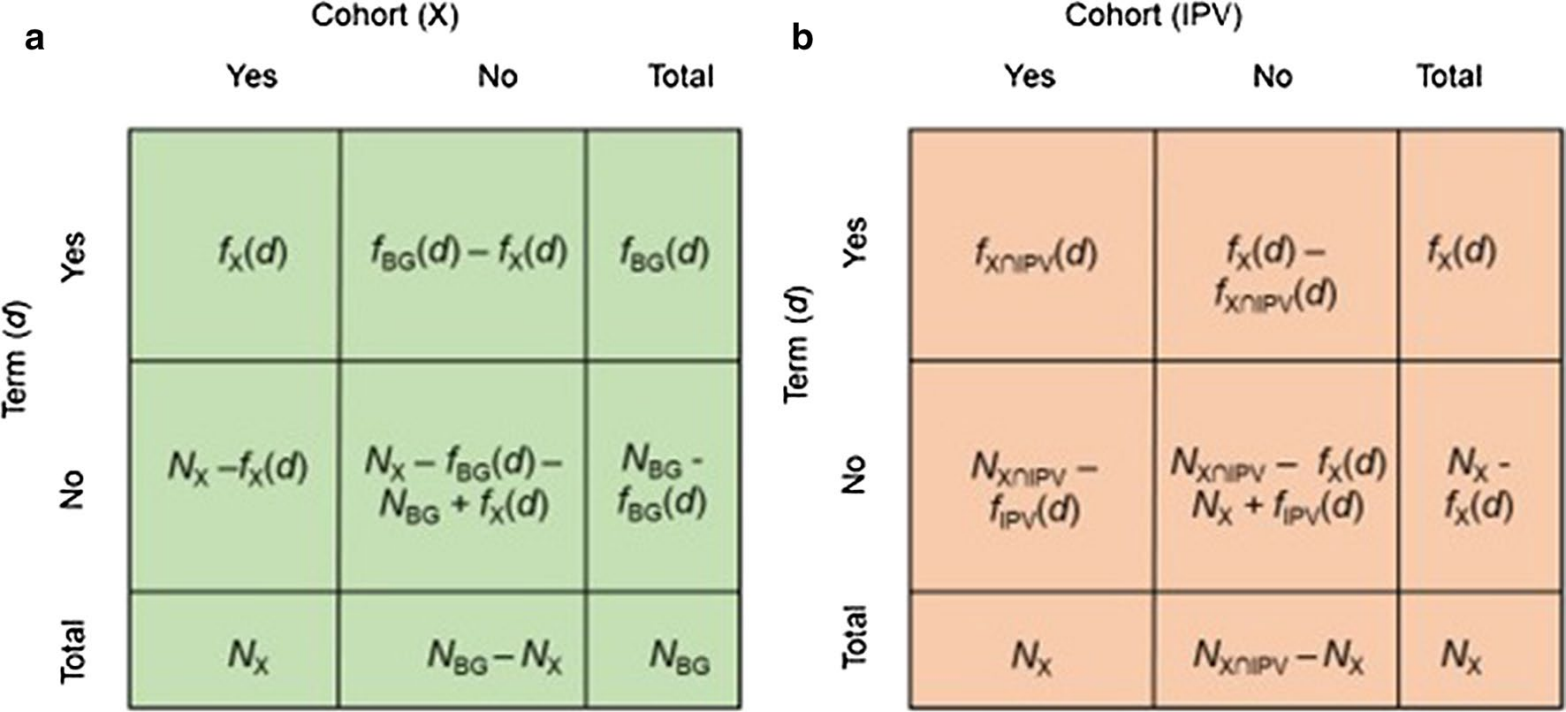
The contingency tables used to assess the significance of the (conditional) prevalence of a diagnostic term in a cohort of interest. **a** The *prevalence* of a term *d* in cohort X as compared to the background cohort (BG).[Yes, Yes] indicates the number of records in cohort *X* that contain term *d*, [Yes, No] indicates the number of records that are not in cohort X but contain term d, [No, Yes] indicates the number of records in cohort X that do not contain term *d*, and [No, No] indicates the number of records that are not in cohort *X* and do not contain term *d*. These entries are computed using the following four statistics obtained from query results: The number of records in the background cohort (*N*_BG_), the number of records in cohort X (N_X_), the frequency of term in the background cohort (*f*_BG_(d)) and the frequency of term *d* in cohort X (*f*_X_(d)). **b** The *conditional prevalence* of a term *d* in the intimate partner violence (IPV) cohort (IPV ∩ X) as compared to cohort X.[Yes, Yes] indicates the number of records in the cohort of records that contain both *X* and IPV, and also contain term *d*, [Yes, No] indicates the number of records that are in cohort X and contain term d, but are not in the IPV cohort.[No, Yes] indicates the number of records that contain both X and IPV but do not contain term *d*, and [No, No] indicates the number of records that are in cohort *X,* do not contain term *d,* and are not in the IPV cohort. These entries are computed using the following four statistics obtained from query results: Number of records in cohort X (N_X_), the frequency of term *d* in cohort X (*f*_X_(d)), the number of records in cohort IPV ∩ X, i.e., those that contain both IPV and X (*N*_IPV∩X_), the frequency of term *d* in cohort IPV ∩ X (*f*_IPV∩X_(*d*))

We call a term *commonly prevalent* in TBI and IPV if it is *significantly prevalent in both* the IPV and TBI cohorts. For a given cohort *X* (*where X* can be the IPV cohort, the TBI cohort, or any of the control cohorts), we consider all terms that have a non-zero frequency in that cohort, i.e., terms that are listed in at least one record in the cohort. To assess the significance of the prevalence of a term in a given cohort, we construct 2 × 2 contingency tables as shown in Fig. 1a. Based on the contingency table for a given term *d* and cohort *X,* we assess the significance of the prevalence of *d* in cohort X using χ^2^ Independence test with Bonferroni correction for multiple hypothesis testing (each term-cohort pair represents a unique hypothesis). However, since sample sizes are very large, the *p *values computed using χ^2^ test may not be suitable for scoring or ranking the terms [28]. For this reason, we also compute the log-odds ratio for a term-cohort pair as follows:

$$\begin{aligned} & {\text{LOR}}\left( {d,{\text{X}}|{\text{BG}}} \right) = \log \left( {f_{{\text{X}}} \left( d \right)} \right) + \log \left( {N_{{\text{X}}} - f_{{{\text{BG}}}} \left( d \right) - N_{{{\text{BG}}}} + f_{{\text{X}}} \left( d \right)} \right) \\ & \quad - \log \left( {f_{{{\text{BG}}}} \left( d \right) - f_{{\text{X}}} \left( d \right)} \right) - \log \left( {N_{{\text{X}}} - f_{{\text{X}}} \left( d \right)} \right). \\ \end{aligned}$$

After we compute the log-odds ratios for each term-cohort pair, we assess the common prevalence of a term in the IPV and TBI cohorts. For this purpose, we define the common prevalence score (CP) of a term with respect to the IPV and TBI cohorts as the minimum of its log-odds ratio (LOR) in the IPV and TBI cohorts, i.e.:

$${\text{CP}}_{{{\text{TBI}}}} \left( d \right) = {\min}\left\{ {{\text{LOR}}\left( {d,{\text{TBI}}|{\text{BG}}} \right),{\text{LOR}}\left( {d,{\text{IPV}}|{\text{BG}}} \right)} \right\}.$$

Defined this way, the common prevalence score of a term *d* is high if and only if *d* is significantly prevalent in *both* IPV and the TBI cohorts. As a control, we also compute a common prevalence score for all terms with respect to all of the control cohorts. Namely, for a given term *d* in cohort *X*, we define the common prevalence score of *d* in IPV and X as:

$${\text{CP}}_{{\text{X}}} \left( d \right) = {\min}\left\{ {{\text{LOR}}\left( {d,{\text{X}}|{\text{BG}}} \right),{\text{LOR}}\left( {d,{\text{IPV}}|{\text{BG}}} \right)} \right\}.$$

Once we compute the common prevalence scores of all terms in the TBI and IPV, as well as the control and IPV cohorts, we systematically compare the distribution of common prevalence scores across different cohorts. To assess the significance of the common prevalence of individual terms in the TBI and IPV cohorts, we estimate thresholds for significance based on the distribution of common prevalence scores in the control cohorts. We describe this process in the Results section.

#### Identification of synergistically prevalent terms

Besides conditions that are commonly prevalent in both the IPV and TBI cohorts, we are also interested in identifying the conditions that are more likely to be seen in patients who are *diagnosed with both IPV and TBI*, although they may not be prevalent in either cohort. We call such terms *synergistically prevalent* terms, since their prevalence in the presence of both IPV and TBI reflects the synergy between IPV and TBI.

To identify synergistically prevalent terms, we first define the *conditional prevalence* of terms. Conditional prevalence refers to the relative prevalence of a term in a cohort when the background population is restricted to a specific cohort (as opposed to the overall population). Namely, to assess the conditional prevalence of a term in cohort X with respect to cohort Y, we compare the frequency of a term in cohort X ∩ Y to its frequency in cohort Y. For this purpose, we use the χ^2^ test and log-odds ratio (LOR) to assess the increase in the frequency of the term when condition X in addition to condition Y is present. We utilize 2 × 2 contingency tables again to assess the conditional prevalence of all terms between our IPV predictor cohort against the eight outcome cohorts. The contingency table used to assess the conditional prevalence of a term *d* in the IPV cohort with respect to cohort X is shown in Fig. 1b. The conditional prevalence of a term in cohort X with respect to the IPV cohort is computed similarly, by setting the sum of the first row to the frequency of the term in the IPV cohort and the table sum to the size of the IPV cohort.

Observe that the strength of a term’s conditional prevalence found in one direction (e.g., IPV|TBI) can be different from the other (e.g., TBI|IPV), since the observation of IPV for a patient with TBI can increase the likelihood of a third condition, while observation of TBI for a patient with IPV may not have any effect on the likelihood of that condition or vice versa. For this reason, we assess the synergistic prevalence of terms with respect to two conditions by examining the significance of these two conditional prevalence scores. Observe that the conditional prevalence of a term in cohort X with respect to cohort Y is proportional to the prevalence of this term in cohort X with respect to the background population (BG), since cohort Y represents a subsampling of the background population. Therefore, to assess the significance of the conditional prevalence of term in cohort X with respect to cohort Y, it is necessary to characterize the relationship between prevalence in the overall population and conditional prevalence in a null model. While it is possible to analytically characterize this relationship, the independence assumptions needed to formulate these relationships may not hold here. For this reason, we use empirical approach terms that have unusually high conditional prevalence with respect to a specific cohort, given their prevalence with respect to the general population.

**Fig. 2:**
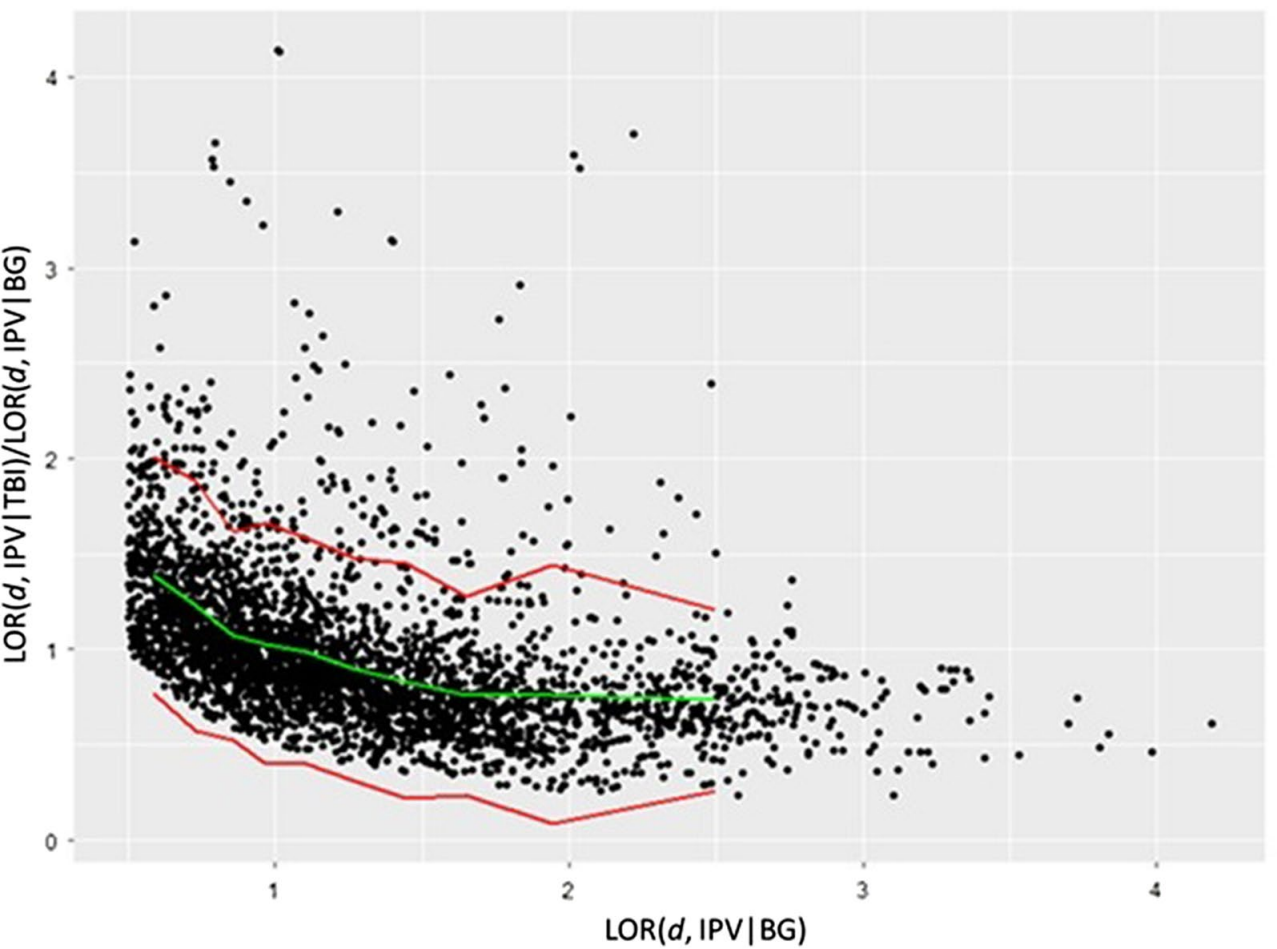
Identification of terms that are significantly conditionally prevalent in the intimate partner violence (IPV) cohort with respect to the traumatic brain injury (TBI) cohort. Each dot represents a term with non-zero frequency in the TBI ∩ IPV cohort (records that have both IPV and TBI as a finding or diagnosis). The log-odds ratio comparing the prevalence of the term in the IPV cohort to that in the background population (LOR(*d*, IPV|BG)) is shown in the x axis. On the y-axis, the conditional prevalence of the term in the IPV cohort with respect to the TBI cohort (LOR(*d*, IPV|TBI)), normalized by the prevalence of the term in the IPV population (LOR(*d*, IPV|BG)), is shown. We divide the terms into 10 bins based on their value on the x-axis. The green curve shows the mean normalized conditional prevalence for the bins, the red curves show the 95% one-sided confidence interval. The terms that lie above the upper red curve have unusually high conditional prevalence in the IPV cohort with respect to the TBI cohort

To empirically identify terms that exhibit the unusually high conditional prevalence (X|Y) given their prevalence in the general population (X|BG), we group terms based on their prevalence in cohort X with respect to the general population (LOR(d, X|BG)). We then assess the distribution of the conditional prevalence of the terms in each group (LOR(d, X|Y)) and construct a 95% one-sided confidence interval based on the observed mean and standard deviation of the conditional prevalence in the group using the Gaussian distribution. We then identify the terms whose conditional prevalence falls outside their group’s confidence interval as terms that have significant conditional prevalence in cohort X with respect to cohort Y. In Fig. 2, this process is illustrated for the identification of terms that are significantly conditionally prevalent in the IPV cohort with respect to the TBI cohort.

## Results

### Shared terms between IPV and TBI

**Fig. 3:**
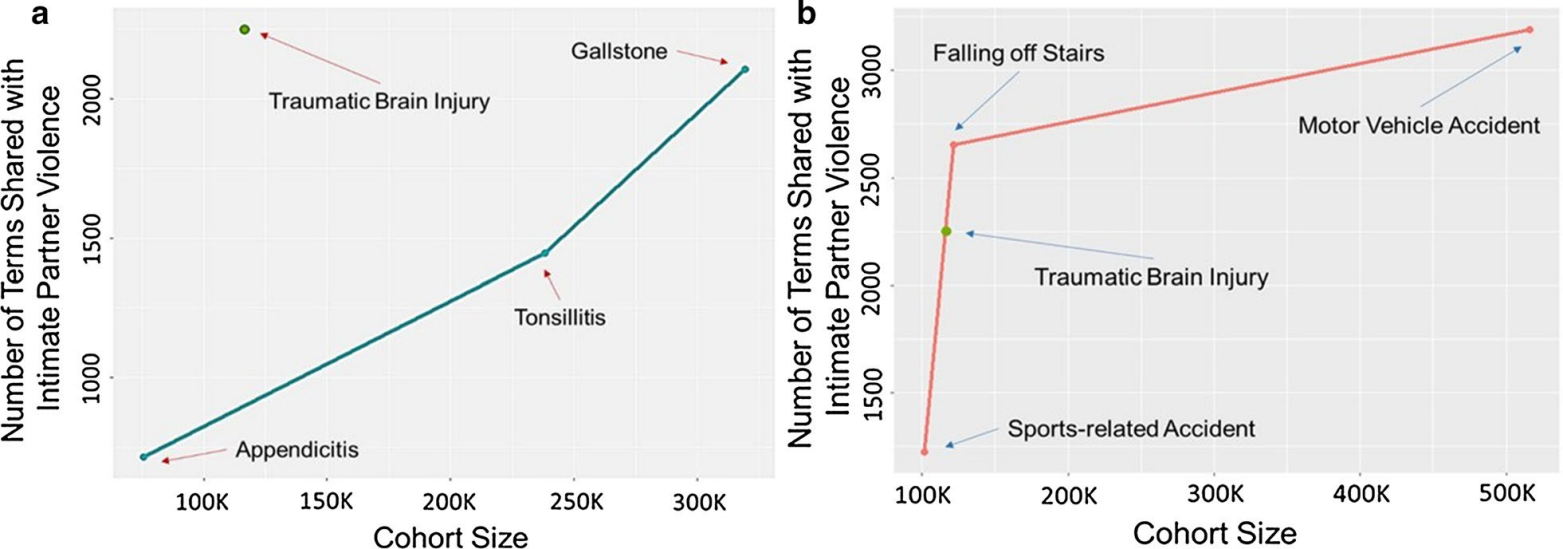
Number of diagnostic terms shared by the intimate partner violence (IPV) and traumatic brain injury (TBI) cohorts, as compared to the number of terms shared by IPV and control cohorts. **a** The cyan line shows the number of terms that have non-zero frequency in both the IPV cohort and the respective acute condition cohort, as a function of cohort size (Left to Right: Appendicitis, Tonsillitis, Gallstones) and **b** The red line shows the number of terms that have non-zero frequency in both the IPV and the respective accident cohort, as a function of cohort size (Left to Right: Sports-related Accidents, Falling off Stairs Incidents, Motor Vehicle Accidents). In both panels, the green dot on both shows the number of terms common to IPV and TBI

The number of terms that have a non-zero frequency in both the intimate partner violence (IPV) and traumatic brain injury (TBI). cohorts are 2,248. To assess the significance of this number, we compare the number of terms that are common to IPV and TBI to the number of terms that are common to IPV and each control condition. These comparisons are shown in Fig. 3.

As seen in Fig. 3a, for acute conditions, the number of terms shared with IPV also grows with cohort size. However, when compared to appendicitis, tonsillitis, and gallstones, TBI has a higher number of shared terms with IPV despite its relatively small cohort size. While the gallstones cohort has nearly three times as many records (319,320) as the TBI cohort (116,660), only 2,107 terms that are present in the IPV cohort are also present in the gallstone cohort. This observation confirms our expectation that there are more shared conditions between TBI and IPV than those that are shared between acute conditions and IPV.

When we compare the number of shared terms between TBI and IPV to the number of shared terms between IPV and the cohorts representing the accident-related control group, we observe a different pattern. The results of this analysis are shown in Fig. 3a. As seen in the figure, the number of terms shared between sports-related accidents and IPV is lower than the number of terms shared between TBI and IPV, although TBI and sports-related accidents have similar cohort sizes. This is not surprising, since sports-related accidents are not usually attributed to IPV. However, when we consider falling off stairs incidents (121,520 records), we observe that the number of terms shared with IPV (2,655) is more than the number of terms shared between IPV and TBI.

When we consider motor vehicle-related accidents, we observe that the number of terms shared with IPV (3,191) is in line with the large cohort size associated with motor vehicle-related accidents (516,040). In other words, with the exception of falling off stairs incidents, the pattern observed in Fig. 3a follows a similar pattern observed in Fig. 3b for the acute conditions control group. These observations suggest that TBI and falling off stairs have more in common with IPV, as compared to acute conditions like tonsillitis, gall bladder, and appendicitis, in addition to accidents including sports-related accidents and motor vehicle accidents. It is also possible that some incidents reported as “falling off stairs” can be associated with IPV.

#### Commonly prevalent terms between IPV and TBI

**Fig. 4:**
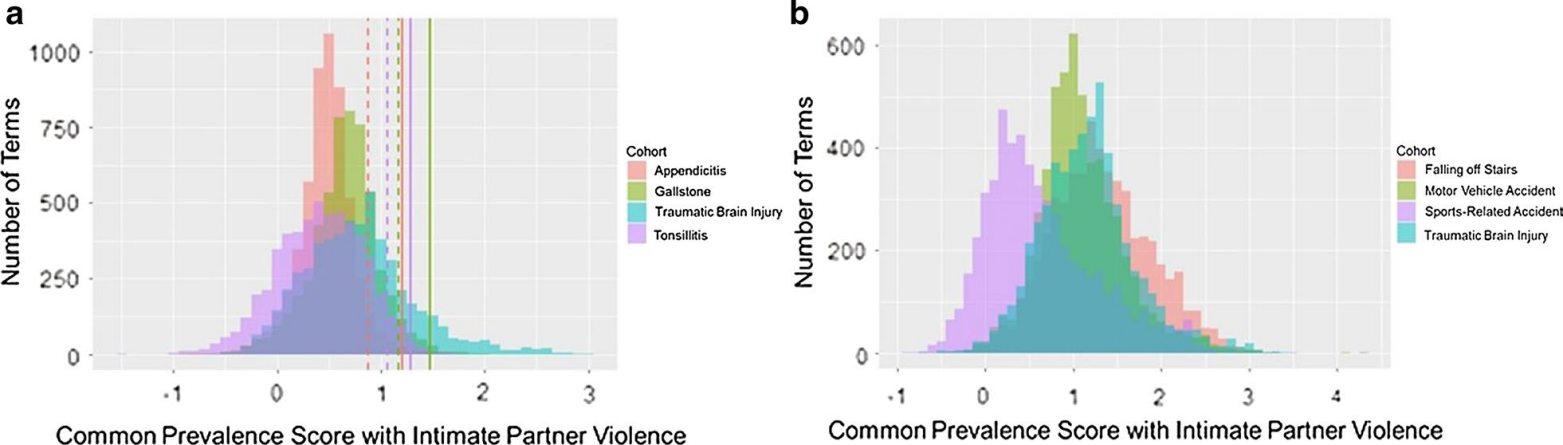
The distribution of common prevalence scores between IPV and TBI cohorts, as compared to the distribution of common prevalence scores between the IPV cohort and each of the control cohorts. For each pair of cohorts, the respective histogram shows the distribution of common prevalence scores across all terms that have non-zero frequency in both cohorts. Common prevalence for a term with respect to two cohorts is defined as the smaller of the two log-odds-ratios of the frequency of the term in each of the two cohorts (thus a term has higher common prevalence if it has a higher log-odds-ration on *both* cohorts). On both panels, the cyan histogram shows the distribution of common prevalence scores for IPV, in comparison to **a** IPV and each of the acute condition cohorts, **b** IPV and each of the accident cohorts. The dashed and solid lines on the left panel show the cut-off lines for a false discovery rate of respectively 5% and 1% based on the acute condition controls, as indicated by the color of the line

For the TBI cohort and each control cohort, we compute the common prevalence scores for all terms that are present in both the respective cohort and the IPV cohort. A term has a high common prevalence score with respect to a pair of cohorts if it is highly prevalent in both cohorts. To obtain a comprehensive view of common prevalence between IPV and each of the other cohorts, we first inspect the distribution of common prevalence scores. The results of this analysis is shown in Fig. 4. As indicated by the clear shift of the cyan histogram in Fig. 4a to the right of the other three histograms, the number of terms that have high common prevalence with respect to the IPV and TBI cohorts is considerably higher than the number of terms that have high common prevalence with respect to IPV and each of the three acute conditions. This observation suggests that terms associated with both IPV and TBI often appear in victims of both conditions. As seen in Fig. 4b, terms shared between IPV and accident-related conditions exhibit a higher common prevalence than the terms shared between IPV and acute conditions. We observe that the common prevalence of terms between IPV and falling off stairs is considerably higher than that between IPV and TBI. The common prevalence of terms between IPV and motor vehicle-related accidents is slightly lower than that between TBI and IPV, and the common prevalence of terms between IPV and sports-related accidents is considerably lower. Overall, these observations suggest that the physical injuries sustained from IPV and subsequent consequences are most similar to the injuries sustained from falling off stairs as compared to other accidents (motor vehicle and sports-related). Furthermore, IPV shares more with TBI than it does with a motor vehicle and sports-related accidents, suggesting that injuries to the head can be relatively more common among victims of IPV as compared to victims of such accidents.

To identify terms that exhibit *significant* common prevalence between IPV and TBI, we decided to use the acute condition control groups as a reference, since the distinction between TBI and the control cohorts was clear for these cohorts. For this purpose, we calculated 1% and 5% false discovery rates (FDR) for tonsillitis, gallstones, and appendicitis based on the distribution of common prevalence scores for each of these cohorts. The respective cut-offs for each cohort are shown in Fig. 4a, with dashed lines representing 5% and solid lines representing 1%. Gallstones have the highest 5% and 1% FDR cut off as compared to tonsillitis and appendicitis. As such, to further reduce the FDR, we call a term significantly commonly prevalent between IPV and TBI at an FDR of 1% or 5% if its common prevalence between IPV and TBI is larger than the respective cutoff for gallstones. At a 5% FDR, there are 1,023 terms that exhibit significant common prevalence between IPV and TBI, while the number of significantly commonly prevalent terms between IPV and TBI is 510 at a 1% FDR.

#### Synergistically prevalent terms between IPV and TBI

**Fig. 5:**
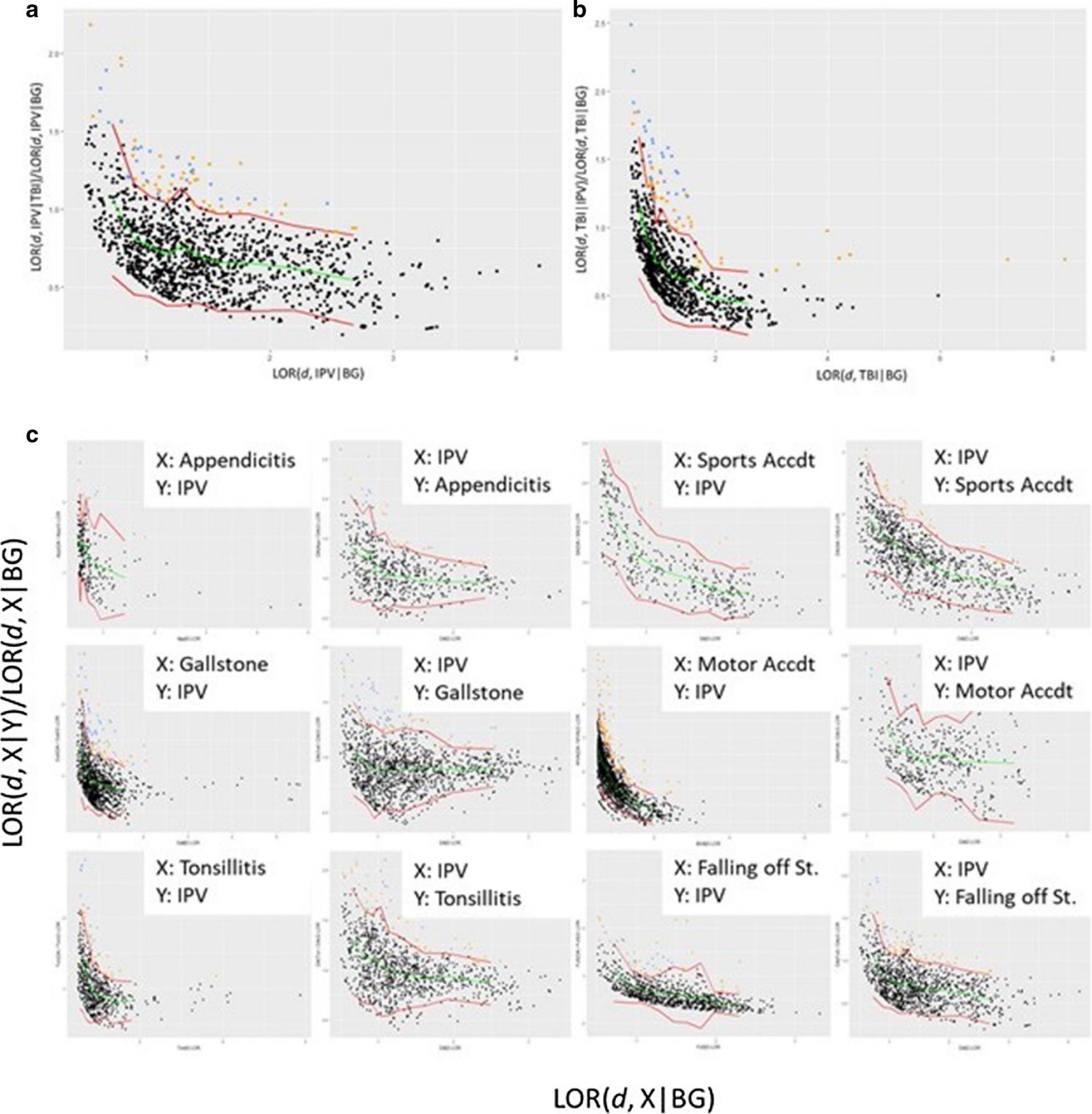
Identification of terms that are synergistically prevalent between intimate partner violence (IPV) and traumatic brain injury (TBI). **a** Terms that exhibit significant conditional prevalence in the presence of IPV among records in the TBI cohort. **b** Terms that exhibit significant conditional prevalence in the presence of TBI among records in the IPV cohort. **c** The assessment of conditional prevalence between IPV and each of the six control cohorts, in each direction. In **a** (respectively in **b**), the scatterplot shows the conditional prevalence of each term in IPV|TBI (TBI|IPV) normalized by the prevalence of the term in the IPV (TBI) cohort, as a function of its prevalence in the IPV (TBI) cohort. The green line represents the mean conditional prevalence as a function of the background prevalence, and the red lines indicate the 95% confidence interval for conditional prevalence as a function of background prevalence. All terms above the upper red line are deemed significantly conditionally prevalent in the respective cohort. The terms that are significantly conditionally prevalent in both directions (IPV|TBI and TBI|IPV) are highlighted in blue, the terms that are significantly conditionally prevalent in only one direction are highlighted in orange. The same coloring scheme is used in **c** as well

The conditional prevalence of the terms shared between TBI and Domestic Abuse (DA) is visualized in Fig. 5. This analysis suggested that there are 81 terms that are significantly conditionally prevalent in the TBI cohort among records that contain IPV as a finding, whereas there are 85 terms that are significantly conditionally prevalent in the IPV cohort among records that contain TBI as a finding. Thirty of these terms are common across both directions. We call these terms *synergistically prevalent* between IPV and TBI, since the term is significantly more prevalent in records that contain both TBI and IPV as a finding, as compared to its prevalence in records that contain only one of these findings. To understand whether the observed synergy between IPV and TBI is statistically meaningful, we also assess the synergy between IPV and each of the control conditions (Fig. 5c).

**Fig. 6:**
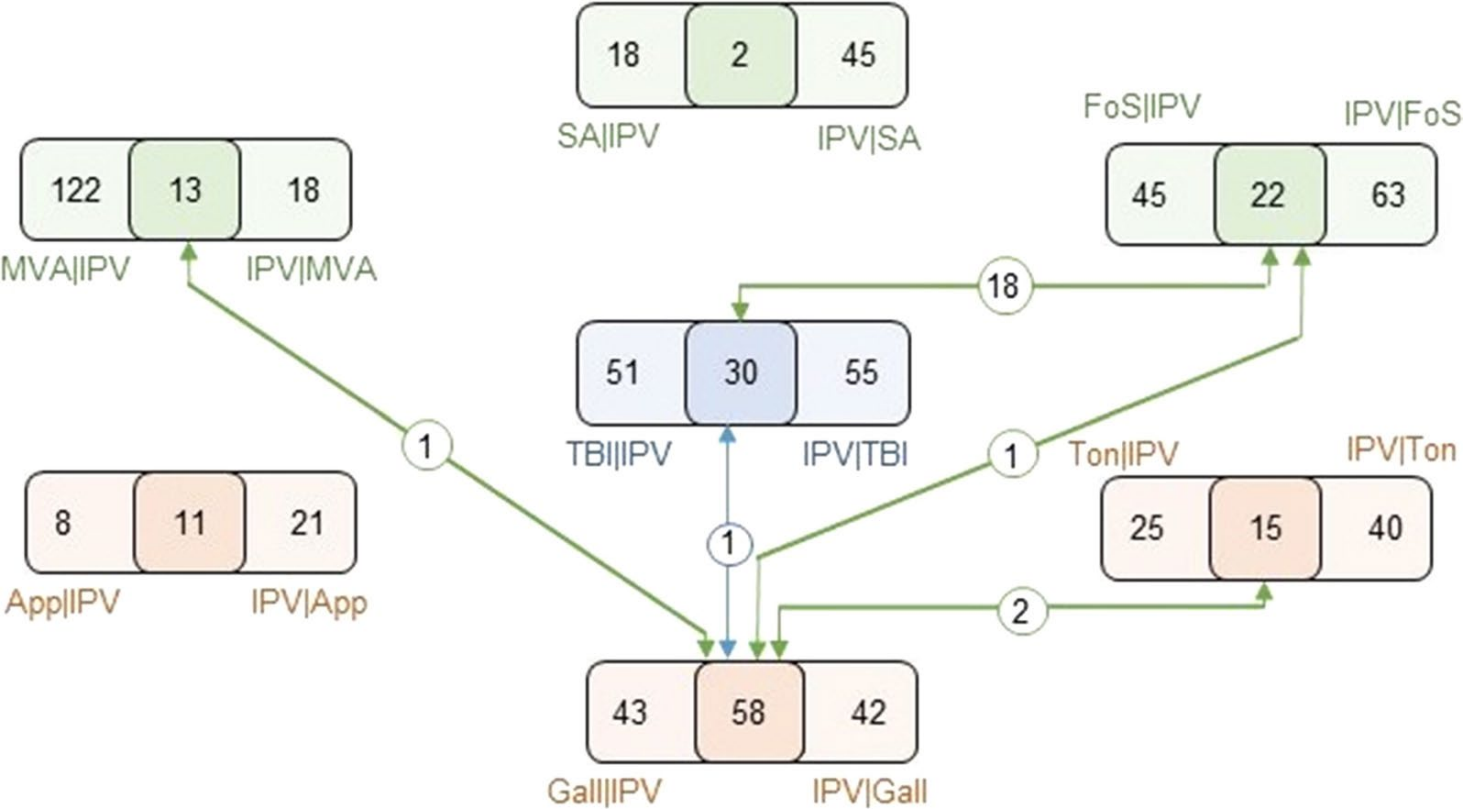
Number of conditionally and synergistically prevalent terms found in each conditional prevalence comparison between intimate partner violence (IPV) and traumatic brain injury (TBI), and each of the six controls, and their intersections. For each condition X, the sum of the numbers in the rectangle on the right shows the number of diagnostic terms that exhibit significant conditional prevalence in the X cohort, when conditioned upon the presence of IPV, the sum of the numbers in the right rectangle shows the number of diagnostic terms that exhibit significant conditional prevalence in the IPV cohort, when conditioned on the presence of X, and the middle square shows the overlap between these “synergistically prevalent” terms. The numbers on the arrows connecting different conditions show the overlap between the synergistically prevalent terms identified for each condition’s comparison to IPV. *SA* sports-related accident, *MVA* motor vehicle accident, *FoS* falling off stairs, *App* appendicitis, *Ton* tonsillitis, *Gall* gallstone

The number of conditionally and synergistically prevalent terms for each control cohort and IPV is shown in Fig. 6. There are many factors that can influence the number of terms that are identified to be associated with a pair of conditions. These factors may include cohort sizes, background statistical variance, confounding factors, and true associations. Since the limitations imposed by the EHR database restrict the number of control cohorts we utilize, we are not able to characterize the background statistical variance for this statistic. For this reason, we compare the number of synergistic terms identified on the six control cohorts against the number of synergistic terms we identify between IPV and TBI. We observe that there are 11 synergistically prevalent terms between appendicitis and IPV, and 15 synergistically prevalent terms between tonsillitis and IPV. Interestingly, there are more synergistically prevalent terms between gallstones and IPV (58 terms) than in TBI and IPV. Our investigation of these terms suggested that these terms were mostly related to various infections and substance abuse. Note that the gallstone cohort also exhibits larger shared prevalence with the IPV cohort as compared to appendicitis and tonsillitis cohorts (in Fig. 4, the histogram that is most shifted to the right after TBI is the green histogram that represents gallstones). This could suggest systematic bias in the data or possible shared confounders between the gallstone and IPV populations. While we were not able to find any reported association between gallstones and IPV, this result may also suggest true associations between these conditions that were not previously reported.

The number of synergistically prevalent terms found between each of the accident-related controls and IPV, namely sports-related accidents (2 terms), motor vehicle accidents (13 terms), and falling off stairs incidents (22 terms) was lower than the number of synergistically prevalent terms found between TBI and IPV. However, for falling off stairs incidents, the number of synergistically prevalent terms found in comparison to the number found in TBI was considerable.

As seen in Fig. 6, there were 22 terms that are synergistically prevalent between IPV and falling off stairs cohorts. Among these, 18 were also synergistically prevalent between TBI and IPV cohorts (Fisher’s exact test *p* value < 0.000001). For the other two accident-related cohorts (motor vehicle accidents and sports-related accidents), however, we observed relatively fewer (respectively 13 and 2) synergistically prevalent terms with the IPV cohort. None of these terms overlapped with the terms that were synergistically prevalent between TBI and IPV. The large overlap between the terms that are synergistic between IPV-TBI and IPV-Falling off Stairs may be indicative of the characteristics of IPV that result in TBI. For this reason, these terms can serve as potential markers for screening for TBI in IPV survivors and/or women who present to the hospital with a falling off stairs incident. The lower number of terms that are synergistic between IPV-TBI and IPV-motor vehicle accidents, on the other hand, may be indicative of the under-reporting of IPV in incidents that involve motor vehicles.

The terms that were synergistic between both TBI-IPV and falling off stairs-IPV included pulmonary, cardiovascular, and excretory complications, as well as burns found on the limbs and hemorrhaging. Leucopenia is unique in that this term is found to be synergistically prevalent in gallstones and IPV, TBI and IPV, and falling off stairs incidents and IPV.

#### Commonly prevalent and synergistically prevalent terms

**Fig. 7:**
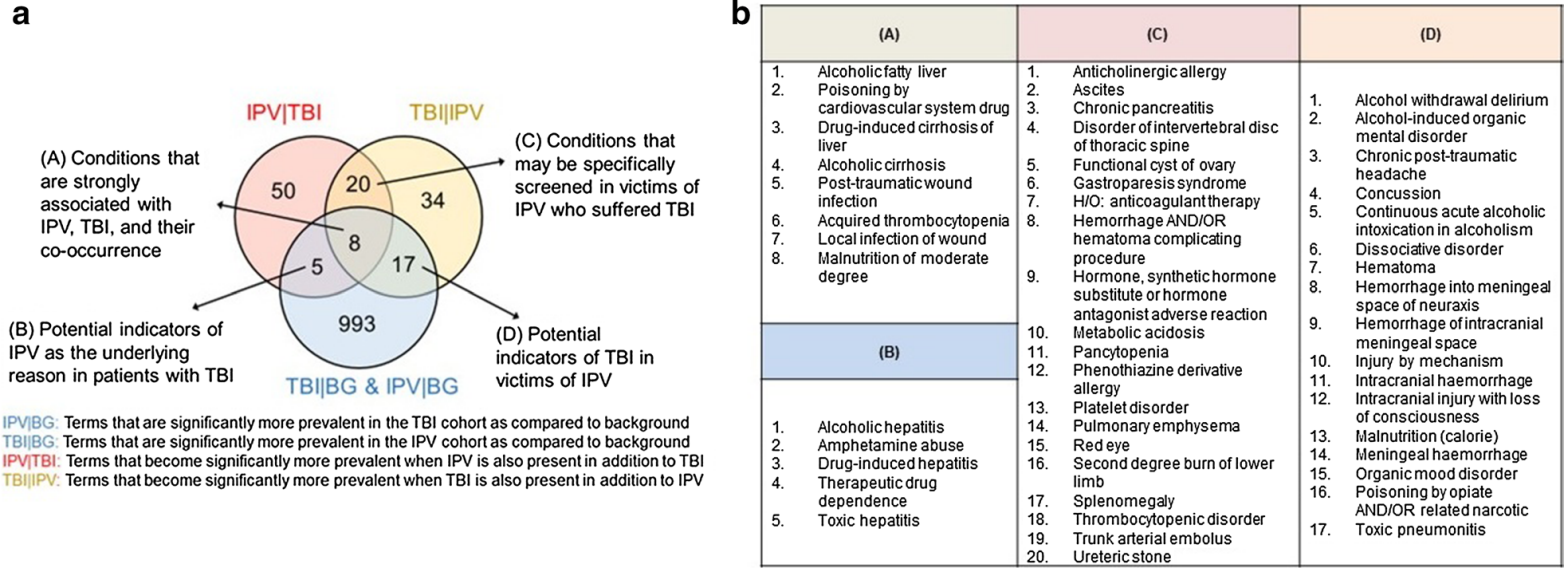
Overlap between commonly prevalent and synergistically prevalent terms between intimate partner violence (IPV) and traumatic brain injury (TBI). **a** Purple and yellow circles represent the significant conditionally prevalent terms in TBI|IPV and IPV|TBI respectively (*p* < 0.05 based on prevalence in the background cohort). The green circle represents the significant commonly prevalent terms in IPV and TBI cohorts (FDR < 5% based on acute conditions control group). The intersections are labeled with their potential clinical implications. **b** The list of terms in each of the intersections

To determine the significant terms found in the interplay between IPV and TBI, we assess the overlap between commonly prevalent and synergistically prevalent terms in IPV and TBI. To identify a set of terms that are commonly prevalent in IPV and TBI, we choose the 5% FDR computed based on the acute control conditions. The results of this analysis are shown in Fig. 7. As seen in the figure, there are eight commonly and synergistically prevalent terms found in both IPV and TBI. Namely, these terms are significantly more prevalent as compared to the overall population in *both* TBI and IPV (hence commonly prevalent), and the prevalence of the term significantly increases if one of TBI and IPV is observed *in addition to the other condition* (hence synergistically prevalent). These eight terms are the following: malnutrition, acquired thrombocytopenia, post-traumatic wound infection, local infection of the wound, poisoning by a cardiovascular drug, alcoholic cirrhosis, alcoholic fatty liver, and drug-induced cirrhosis. The odds ratio for the occurrence of these terms in the comparison of IPV and TBI cohorts to the background cohort, as well as to each other, are shown in Table 2.

**Table 2:** The odds ratios for the terms that are commonly prevalent and synergistically prevalent in traumatic brain injury (TBI) and intimate partner violence (IPV) (the intersection labeled (a) in Fig. 7)

Term	IPV|BG	TBI|BG	IPV|TBI	TBI|IPV
Alcoholic fatty liver	17.2	6.6	12.9	6.3
Poisoning by cardiovascular system drug	9.6	6.2	7.4	6.3
Drug-induced cirrhosis of liver	11.0	5.7	7.0	4.2
Alcoholic cirrhosis	11.0	5.7	7.0	4.2
Post-traumatic wound infection	6.7	5.5	5.8	6.3
Acquired thrombocytopenia	5.8	5.0	5.6	6.3
Local infection of wound	7.3	5.4	5.5	5.1
Malnutrition of moderate degree	5.5	5.4	4.8	6.3

Odds ratios are shown for the frequency of these terms in the IPV cohort compared to the background cohort (IPV|BG), TBI cohort compared to the background cohort (TBI|BG), IPV and TBI cohort compared to the TBI cohort (IPV|TBI) and IPV and TBI cohort compared to the TBI cohort (TBI|IPV)

## Discussion

### Synergistic terms

Our findings suggested that health effects attributed to malnutrition, acquired thrombocytopenia, post-traumatic wound infection, local infection of the wound, poisoning by a cardiovascular drug, alcoholic cirrhosis, alcoholic fatty liver, and drug-induced cirrhosis are highly significant at the joint presence of intimate partner violence (IPV) and traumatic brain injury (TBI). To this end, it is somewhat surprising that these terms are not directly attributed to TBI by prior studies. However, these terms likely represent the adverse health effects of both IPV and TBI, as well as the conditions that make women more vulnerable to IPV and particularly assault to the head. Liver cirrhosis and observed fatty liver due to alcohol and substance abuse have been previously observed in IPV victims. Alcohol and drug consumption is often used as a coping mechanism for IPV victims suffering post-assault [29–32]. Furthermore, alcohol consumption may increase vulnerability to experiencing IPV [29–32]. The presence of malnutrition among these terms suggests that victims of IPV also have a higher likelihood of suffering from anemia and being underweight compared to non-IPV victims [33]. The presence of wound infections suggests that physical injuries frequently occur in victims of both IPV and TBI, but the location of these wounds is not directly referenced. A term that is relatively unexpected among these eight terms is thrombocytopenia, which has an association with pregnancy complications [34]. However, it is also reported as a side-effect exhibited by severe TBI [35]. In this respect, thrombocytopenia may indeed be indicative of the synergy between IPV and TBI, in that pregnant women are also more vulnerable to assaults by their intimate partners.

### Commonly prevalent terms

There are 17 terms that were commonly prevalent in both TBI and IPV cohorts, and are conditionally prevalent in TBI|IPV, i.e., the likelihood of observing these terms increased significantly when TBI was observed in addition to IPV. These conditions ranged from a concussion, chronic post-traumatic headache, hematoma, various types of cranial hemorrhages, alcoholic and drug-related abuse and poisoning, delirium, pneumonia, and caloric malnutrition. Most of these conditions were also reported by prior studies as adverse health effects of TBI [7, 8, 11]. These terms can be useful as potential indicators of TBI in victims of IPV.

### Relationship to strangulation

Although strangulation is not used as a term in *Explorys*, many terms that we found as significantly prevalent in both TBI and IPV cohorts were significant to the neck area of a patient, thus could imply that the injury can be related to strangulation or asphyxiation. In forensic literature asphyxia is composed of four key groupings including suffocation, strangulation, mechanical asphyxia, and drowning [36]. The terms that were related to neck injuries include ‘Whiplash injury to neck’, ‘Fracture of neck of metacarpal bone’, ‘Neck sprain’, ‘Strain of neck muscle’, ‘Sequelae of injuries of neck and trunk’.

### Synergistic and commonly prevalent terms

We identified five terms that are commonly prevalent in both TBI and IPV, and are conditionally prevalent in TBI|IPV. The likelihood of observing these terms increases significantly when IPV is observed in addition to TBI. For this reason, we annotated these terms as “Potential indicators of IPV as the underlying reason in patients for TBI”, since these terms can be used to provide medical providers with pointers while screening for IPV in patients with TBI. These conditions mostly relate to drug abuse, including hepatitis due to drug or alcohol abuse. This set of conditions were perplexing since they mostly focus on additional liver-related damages. Interestingly, it has been reported that patients who have experienced TBI are at a higher risk of acquiring liver cirrhosis due to intracerebral hemorrhaging [37]. Subsequently, the risk of acquiring liver cirrhosis or complications to the liver is high from increased alcohol or drug consumption [29–32]. This suggests that liver cirrhosis and any subsequent complications of the liver could be a strong indicator of IPV-related TBI, in addition to substance and alcohol abuse. In other words, if a patient who has experienced TBI also exhibits signs of any adverse liver conditions from drug or alcohol abuse, it may be likely this patient also suffered from an IPV-related event.

### Potential impact on clinical practice

Our analyses resulted in the identification of four sets of medical findings/terms that can inform clinical practice or further research. These sets are illustrated and the terms in each set are listed in Fig. 7. We interpret these sets as follows:

- *Potential indicators of IPV as the underlying reason for patients with TBI* We found that the likelihood of observing this term in a record increased significantly if the record included IPV in addition to TBI, as compared to all records that had TBI. Thus, it follows by the fundamental theorem of Bayesian statistics that the presence of these conditions in a patient who presents with TBI would increase the likelihood that the patient is subjected to IPV.
- *Potential indicators of TBI in victims of IPV* We found that the likelihood of observing this term in a record increased significantly if the record included TBI in addition to IPV, as compared to all records that had IPV. Thus the presence of these conditions in a victim of IPV would increase the likelihood that the patient may have suffered TBI.
- *Conditions that may be specifically screened in victims of IPV who suffered TBI* The likelihood of these terms increased with the observation of TBI/IPV in addition to IPV/TBI in both directions, thus these terms are identified as strongly synergistic with the co-occurrence of IPV and TBI. Thus, for victims of IPV who suffered TBI, these terms can be screened for preventive purposes.
- *Conditions that are strongly associated with IPV, TBI, and their co-occurrence* In addition to being synergistically associated with the co-occurrence of IPV and TBI, these terms were also significantly prevalent in each of IPV and TBI cohorts. Thus these terms can be screened for victims of IPV or patients with TBI regardless of the presence of TBI/IPV.

#### Screening for TBI in IPV victims

TBI sustained due to IPV often occurs over time and ranges in severity; it can be easily overlooked by both victims and health care professionals [38]. Therefore, it is critical to provide assessment and treatment to ensure the well-being of victims of IPV. In standard practice, symptoms that emerge following head trauma are assessed through standardized and non-standardized measures. For example, the Glasgow Coma Scale [38] is frequently used to identify the extent of consciousness based on the eye-opening, motor, and verbal response after the head trauma. Scoring the degree of the symptoms into severe, moderate, and mild TBI (mTBI). With this observational assessment, it is difficult to identify the mild TBI cases due to a lack of loss of consciousness [39]. In addition, it is challenging to identify mild cases via imaging [39]. In 1993, experts from Rehabilitation Medicine published a definition of mTBI further detailed the diagnostic categories for mTBI. According to this definition, any period of loss consciousness 30 min or less, loss of memory, altered mental state, and focal neurological deficits without being identified as severe or moderate cases, can be considered as mTBI [40]. The effects of mTBI are more likely to be observed at the cellular and vascular levels as a result of the structural and functional status of the central nervous system [41].

The terms listed in Fig. 7 can be helpful for further investigation of potential TBI among victims of IPV. In particular, these terms can also help clinicians further distinguish Post Traumatic Stress Disorder (PTSD) from TBI. Distinguishing TBI from PTSD symptoms can be challenging since these two conditions share many common symptoms [42]. Health care providers can also ask more direct questions that might be indicative of potential TBI causing events with loss of consciousness, blacking out, or seeing stars, particularly if IPV is suspected.”

#### Limitations

The purpose of the data mining framework we apply here is to identify statistically significant patterns that can be used to gain insights into the interplay between IPV and TBI. It is important to note that, as in any data mining application, the identified patterns may not represent true associations despite their significance. To this end, while the results we present here provide potentially useful pointers for further research, they need to be validated by additional controlled studies before they can be used to guide clinical practice.

In addition to the general limitations of data mining, there are few constraints that pose limitations to our analyses and results. One major limitation is the utilization of SNOMED-CT and IC codes to define intimate partner violence cohorts. Healthcare providers use ICD codes for domestic violence quite infrequently. This limits our ability to reach a variety of IPV instances. It is possible that we were only accessing IPV cases in high severity that cause injury and ailment while missing out on less severe violence instances and in turn subtler symptoms. Infrequent use of ICD codes might also be related to survivors' efforts to avoid drawing attention to their abuse, as well as their distrust to providers and the healthcare systems in general. The integration of EHR data with data that is collected outside of healthcare settings can be effective in addressing this issue. Another strategy that can potentially enable identification of IPV instances in EHR data can be to use terms that are associated with IPV. The significant terms that are identified in this study can provide a useful pointer for such efforts. However, validation of these potential markers requires the availability of EHR data in higher resolution than the summary data we used in our analyses.

Another limitation of the study is that the data we were able to access through the IBM *Explorys* platform is HIPAA de-identified data. The only raw metric for each term for all of our data-mined cohorts are the frequency of occurrence of each term. As a result, we do not have the dimension of time for each condition attributed to the record. Therefore, without accessing the time aspect from the data set, the overall representation of IPV and TBI in that context is underrepresented within the entire EHR population.

Finally, non-reported IPV incidents within the electronic health records (EHRs) pose important challenges to the analytical framework we develop here. IPV incidents maybe not reported by victims due to legal implications that could impact the victims. As a result, the EHRs analyzed in this study may not fully represent all possible IPV cases that could be mined and analyzed. However, our findings can assist in making inferences of a patient’s injuries and trauma that could be caused by IPV.

## Conclusion

Results from our analyses will assist in the development of new hypotheses on the interplay between intimate partner violence (IPV) and traumatic brain injury (TBI), as well as their potential health consequences. While our statistical assessment was rather conservative due to the limited access to more detailed metrics from *Explorys* (such as a dimension of time when patients diagnosed with each condition and every record extracted represents a unique patient), we have identified a considerable number of conditions that are associated with IPV, TBI, and/or their co-occurrence. As indicated in our results, common prevalence and synergistic prevalence have different interpretations. This difference can be used in clinical practice to utilize the terms identified with each other for a different clinical purpose. Specifically, our results generated potential markers for the existence of head trauma in IPV victims, markers for the existence of IPV in patients with TBI, and conditions that can be screened more carefully in victims of IPV who suffered head trauma. Therefore, the results presented here can potentially improve the accuracy and confidence of existing clinical screening techniques on determining IPV-induced TBI diagnoses from victims. These results can provide a starting point for developing a versatile and predictive tools that assists clinicians in diagnosing IPV-induced TBI to a victim.

To develop a better understanding of how IPV is related to health of victims, it is useful to explore the interactions among symptoms. Analyzing these relationships may help us discover what physiological systems are more closely associated with experiencing severe consequences of IPV, and could lead to future research into the effects of IPV on the health of victims. Our analysis may help with future research in identifying associations between conditions that are thought to be independent. Health care providers can use this information to improve the prescription of effective treatment preventions and identify trends across populations as well as development of more effective screening.

## Data Availability

This study uses data from IBM *Explorys* Electronic Health Record Database and access to raw data requires approval from IBM *Explorys*. The complete list of the statistical results presented in this manuscript is available upon request from the authors.

## Abbreviations

IPV: Intimate partner violence
TBI: Traumatic brain injury
HER: Electronic health records
SNOMED-CT: Systematized Nomenclature of Medicine Clinical Terms
ICD-9: International Classification of Disease Coding
DA: Domestic abuse
MVA: Motor vehicle accidents
SA: Sports-related accidents
FoS: Falling off stairs
